# Eliciting the experiences of the adolescent-parent dyad following critical care admission: a pilot study

**DOI:** 10.1007/s00431-018-3117-y

**Published:** 2018-02-21

**Authors:** Dora Wood, Sophie Geoghegan, Padmanabhan Ramnarayan, Peter J. Davis, John V. Pappachan, Sarah Goodwin, Jo Wray

**Affiliations:** 1 Paediatric Intensive Care Unit, Bristol Royal Hospital for Children, Bristol, BS2 8HW UK; 2 Centre for Outcomes and Experience Research in Children’s Health, Illness and Disability, Great Ormond Street Hospital, WC1N 3JH, London, UK; 3 Children’s Acute Transport Service, Great Ormond Street Hospital, WC1N 3JH, London, UK; 4 Department of Paediatric Intensive Care, University Hospital Southampton, Tremona Road, Southampton, SO16 6YD UK; 5 Critical Care and Cardiorespiratory Division, Great Ormond Street Hospital, WC1N 3JH, London, UK

**Keywords:** Adolescent medicine, Critical care, Critical illness, Intensive care units, Intensive care units, paediatric, Qualitative research

## Abstract

Critically ill adolescents are usually treated on intensive care units optimised for much older adults or younger children. The way they access and experience health services may be very different to most adolescent service users, and existing quality criteria may not apply to them. The objectives of this pilot study were, firstly, to determine whether adolescents and their families were able to articulate their experiences of their critical care admission and secondly, to identify the factors that are important to them during their intensive care unit (ICU) or high dependency unit (HDU) stay. Participants were 14–17 year olds who had previously had an emergency admission to an adult or paediatric ICU/HDU in one of four UK hospitals (two adult, two paediatric) and their parents. Semi-structured interviews were conducted with eight mother-adolescent dyads and one mother. Interviews were transcribed and analysed using framework analysis.

*Conclusion*: The main reported determinant of high-quality care was the quality of interaction with staff. The significance of these interactions and their environment depended on adolescents’ awareness of their surroundings, which was often limited in ICU and changed significantly over the course of their illness. Qualitative interview methodology would be difficult to scale up for this group.

**Table:** 

**What is known** • *Critically ill adolescents are usually treated on intensive care units optimised for older adults or younger children.* • *The way they access and experience health services may be different to most adolescent patients; existing quality criteria may not apply.*
**What is new** • *Reported determinants of high-quality care were age-appropriateness of the environment, respectfulness and friendliness of staff, communication and inclusion in healthcare decisions.* • *The significance of these depended on adolescents’ awareness of their surroundings, which was often limited and changed over the course of their illness.*

## Introduction

After decades of being managed in healthcare settings either as large children or as small adults, there is increasing recognition that adolescents are a distinct patient group with unique physical, psychological and behavioural needs [11]. Attention has now turned to making health services fit for purpose for adolescents with the publication of quality criteria [1, 5, 16] and development of patient-centred quality indicators [2].

In the United Kingdom (UK), around 4500 critically ill adolescents (12–19 years old) are admitted to either adult or paediatric intensive care units (ICUs) each year [19]. The way critically ill adolescents access and experience health services may be very different to the majority of adolescent service users: many of the choices about location of care or service provider are not available to them, and the effects of drugs and critical illness may render them less able to participate in decisions about their care. Efforts have been made to tailor the delivery of intensive care for critically ill adolescents and their families [17], but their opinions have rarely been sought, despite the growing emphasis on the importance of patient experience and the patient voice in health policies. However, there are likely to be specific challenges in elucidating such information.

The objectives of this pilot study were firstly to determine whether adolescents and their families were able, through semi-structured interviews, to articulate their experiences of an admission to an ICU or high dependency unit (HDU) and secondly to identify the factors that are important to them during their ICU/HDU stay.

## Materials and methods

The study was grounded in the principle that young people should have a voice and agency in their own healthcare [18]. Parents were also included as they are critical observers of their child’s care in ICU/HDU. Eligible participants for this study were (1) adolescents aged 14–17 years who had been admitted as an emergency to an adult or paediatric ICU or HDU in one of four UK hospitals (two adult, two paediatric) for at least 24 hours in the previous 12 months, were at least 2 months post-ICU admission and were awake for some of their stay in ICU and (2) their parents/carers. Local specialist nurses at each participating hospital contacted all eligible patients and their families to seek their consent to be contacted by a researcher and invite them to take part in an interview. Participants were provided with opportunities to discuss the research further, after which written informed consent was obtained from all participants (and from parents of those aged under 18 years) prior to commencing interviews.

All interviews were conducted face-to-face by a single researcher (a female social scientist with experience of interviewing intensive care patients and their families) and took place in families’ own homes or in quiet rooms across the hospital. Interviews lasted 30–90 min and were audio-recorded and transcribed verbatim. Topic guides were used to provide general structure, although participants were encouraged to influence the direction of interviews. Participants were asked to recall what they could remember of their admission to ICU and the care and support provided by staff. The researcher made contemporaneous notes of the individual sessions and her reflections on the process which were reviewed with other team members.

Qualitative data were analysed using the Framework approach [15], a process involving five distinct though highly interconnected stages: familiarisation, identifying a thematic framework, indexing, charting, mapping and interpretation. It allows themes to develop from both the research questions and narratives of research participants [13]. During the analysis, a series of “frameworks” or grids were constructed, into which the summarised qualitative data were entered under descriptive headings. Two members of the research team (a psychologist and an intensive care consultant, both of whom have significant clinical and research experience of intensive care), together with the researcher, independently (to enhance credibility) generated and agreed these descriptive headings after careful reading of the transcripts. A flexible, iterative approach to this phase of the analysis process was adopted, with descriptive headings being refined as indicated through regular discussion with team members. Data from each transcript were individually entered into the framework, and key themes were extracted from the completed frameworks and the relationships between the themes explored. The data and findings were discussed with other team members and two experienced intensive care nurses who were not directly involved with the project to enhance confirmability and credibility of the findings.

Ethical approval was granted from the South West—Central Bristol ethics committee (Ref 14/SW/1131).

## Results

### Feasibility

#### Recruitment

All 14 families identified as fulfilling all of the inclusion criteria during the recruitment period agreed to be contacted by the research team. Eight mother-adolescent dyads participated in interviews, and one mother was interviewed alone. Of the remaining five families, three could not be contacted after the initial agreement to speak to the researcher, one agreed to participate but for logistical reasons could not be recruited in the time-frame of the study and one agreed but then cancelled. Table 1 gives a summary of participants and a brief description of the details associated with their admission. To avoid identification of participants, direct quotes used to illustrate findings will be attributed to adolescent (teenage) participant (T), parent participant (P), adult services (A) and children’s services (C).

**Table 1:** Case-by-case summary of participants

Participants	Age of patient (at interview)	PICU or AICU	Details
P1	17	AICU	Admitted via emergency department—sudden acute life-threatening condition. Discharged via ward; no on-going treatment required.
T2 and P2	15	PICU	Admitted via ward—part of inter-current illness. On-going treatment required.
T3 and P3	16	PICU	Admitted via emergency department—part of on-going, lifelong condition. Discharged via ward; on-going treatment required.
T4 and P4	17	AICU	Admitted via emergency department—sudden acute life threatening condition. Discharged via ward; no on-going treatment required.
T5 and P5	15	PICU	Admitted via emergency department at local hospital—sudden acute life-threatening condition. Discharged via ward; on-going treatment required.
T6 and P6	14	PICU	Admitted via ward—newly diagnosed inter-current illness. Discharged back toward; on-going treatment required.
T7 and P7	15	PICU	Admitted from local hospital via emergency department—sudden acute life threatening condition. On-going treatment required.
T8 and P8	19	AICU	Admitted via ward, critical care required during procedure. Discharged via ward—will have on-going contact with hospital
T9 and P9	16	AICU	Admitted via emergency department—sudden acute life threatening condition. Discharged via ward; on-going condition.

#### Ability to report on the intensive care experience

Two particular features noted in the interviews influenced the information adolescents provided about their ICU admission. Firstly, although interviews were focused on the ICU admission, all participants discussed their experience of being in hospital in its entirety. The discussions could not be limited to one element of hospitalisation—rather, the hospital experience is holistic.

Secondly, as predicted, participants often had impaired awareness during at least part of their ICU or HDU stay. During interviews, some participants acknowledged that they remembered little of their experience of receiving critical care: recollections for some participants amounted to waking up and being moved to another ward shortly afterwards. In some cases, it was evident that participants were recounting what they had heard from their parents and some sought confirmation from their parent during interviews.

### Factors contributing to intensive care experience

#### Environment

Some participants reported that the environment of ICU is less important when you are acutely unwell. “I don’t think it matters, you know, when somebody’s on a ventilator. Psychologically, if they were awake it might matter, because it’s all pretty open” (P1A) and “I think when you’re that ill you don’t care really, and because the nurses there are very hands on, very talkative anyway…I don’t think it would really matter so much if you were on a children’s ward” (T4A).

Some discussed the influence of other patients and age-appropriateness of the environment: “It’s like a dying ward...it was full of very old people, they were on their last legs...they knew they weren’t going to survive long and then they put me in a room like that and one man did start going weird one night” (T9A). Others described activities on a paediatric ward as “childish”, and one reflected: “I wouldn’t mind if people were like fourteen, fifteen, because like, that’s close enough to my age for me to deal with, but if everyone’s like eight, nine, ten, that’s quite young. That’s a lot different than my age. That would be weird” (T4A). Those participants who had experienced an adolescent ward reviewed it positively, but an adolescent unit was not available in any of the ICUs.

#### Staff behaviour

**Fig. 1:**
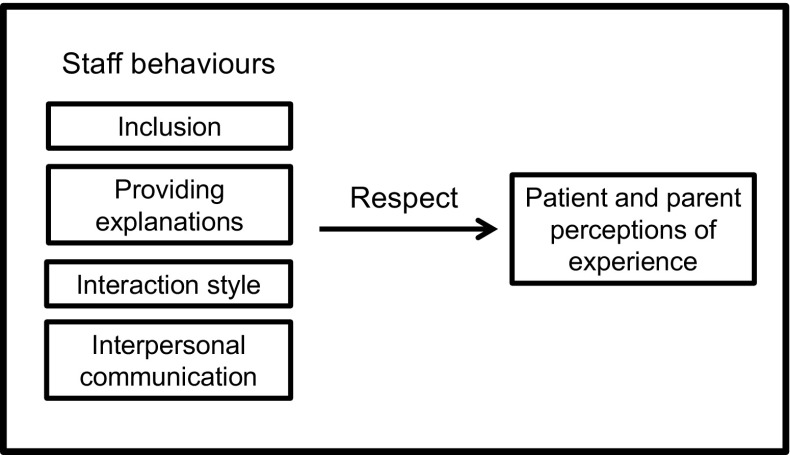
Staff behaviours and their relationship with patients’ and parents’ perceptions of experience

Staff behaviours were a central part of both parents’ and adolescents’ hospital experience. Neither adolescents nor their parents spoke very much about the clinical care they received. Discussion about the technical skills and medical expertise of staff was limited, with most participants referring to it more generally: “care was good”. Discussions centred on care provider behaviour, regardless of whether care was received in a child or adult setting. Data indicated five “key behaviours”, as shown in Fig. 1.

##### Inclusion

Participants discussed the importance of young people themselves feeling involved and included in their own journey and experience, and this was particularly mentioned by users of paediatric services. One adolescent expressed her frustration when staff stood outside her room to discuss her: “I’m sorry but even on this ward, I have a conversation with the doctors to know what’s going on. I’m just meant to sit there and watch you talk about me” (T6C). Another explained: “They talked to mum a bit, or to the nurses, but they would never tell me anything” (T7C). When adolescents were included, they reflected positively on this experience: “I do find it pretty good that I know what’s going on with my body. I know what part of my brain’s functioning” (T3C) as did parents: “Just include the patient…discuss it openly….I think it’s a big deal” [P1A].

##### Providing explanations

Both adolescents and parents discussed the value of being given information and having things explained to them. Adolescents and parents described poor experiences where this did not happen: “there were a couple of times that I would feel they were coming, and they were more brief than in-depth. They wouldn’t give that much information” (T5C), “I feel like I would have done better in an adult ICU … they listen to you whereas in children’s they treated me as if I was actually in a coma” [T6C], or “They tended to treat [her] like she wasn’t with it at times and not explain what they were doing” (T4A). More positively, some reflected: “They [staff] were really good, they would tell you, and if you weren’t sure, you would just ask, they were really good at explaining things” (P3C) “...they kept you up-to-date all the time” (T3C).

##### Interpersonal communication

Adolescents described how they wanted staff to make an effort to get to know them and communicate with them beyond the medical context. For example: “We did have quite funny chats, we played music, sing along to the radio. They did treat me more kind of like a friend” (T2C)…“There are people there, they’ll come round like every hour, say, ‘Hello’ to you, ‘what are you doing?’ and stuff...they can be just nice people rather than just saying, ‘I am doing your bloods’, ‘I am going’” (T7C).

##### Tailoring their communication and interaction style

A number of participants discussed being ‘treated’ either as an ‘adult’ or a ‘child’ referring to how their care providers tailored their communication and interaction style. Pitching communication in a more adult way was received positively*:* “They knew that I was quite grown up, mentally, and I knew exactly what was going on, so then they started treating me like an adult, even though I was on a children’s ward...I had proper conversations, and we proper talked about treatment as if I were an adult, and I really enjoyed that” (T2C)… “… as he’s got older, they talk to him more like an adult now” (T3C). In contrast, some participants reflected negatively that they felt they were treated either like a young child—“they treated me like a five year old. I really didn’t like it” (T6C)… “I think that they don’t realise now that 14 year old kids are quite old in their years really, they’re not quite adults, but they know what’s going on a lot more than they give them credit for” (P6C) or an older person: “I’m not old, I’m X years and I am with it, I understand everything that’s going on, could you just explain it to me…the adult ward, they only really know how to treat people who are quite old and on the children’s ward, I think they’d probably treat you quite young…that’s the problem. You’re stuck in the middle” (T4A).

Several participants described how adolescents are their own distinct group and require a different interaction style to adults and children: “There was that space in between where she needed information at [her] level but she didn’t need it as a child, but she didn’t need the full impact of an adult either, she just needed to get the facts” (P7C).

##### Respect

It was evident that perceptions of staff behaviours impacted on whether adolescents felt they were respected. One participant explained “I had to have a...line put in...pushed in very hard, she put me in pain and I said, ‘I’m in pain’ and she was there trying to say, ‘Don’t worry’...I wasn’t listened to the whole time I was there” (T6C). Another reported “…this one doctor came in and brought in like three or four students with him, I think they were students...I had no idea, I didn’t know who the doctor was, I didn’t know who any of the students were, obviously and they just came in and just started talking to me and doing things and I was like, who the hell is this person? I got very confused...So after that, my Mum spoke to them and told them not to do that anymore” (T4A).

Overall, *where* care was received was perceived as less important than *how* care was provided: “I think it’s more how the nurses actually speak to you rather than where you are or anything like that…” (T4A).

## Discussion

We set out to address two related questions: firstly whether adolescents and their families were able, through semi-structured interviews, to articulate their experiences of an admission to an ICU/HDU and secondly to identify what factors are important to them during that admission.

Despite the perceived importance of an appropriate physical environment and age-appropriate facilities, the main reported factor contributing to the quality of the healthcare experience in our study was the quality of interaction with staff. The significance of these interactions and their environment depended on adolescents’ awareness of their surroundings, which was often limited in ICU and changed significantly over the course of their illness [12]. Our methodology was effective in elucidating these themes but would not be feasible to scale up to a significantly larger sample size.

Adult and paediatric intensive care units are, understandably, designed around the needs of the majority of their patients. However, these efforts may not suit critically ill adolescents; we know that healthcare priorities differ between younger and older children [3] and also between younger and older adults [6]. Critically ill adolescents and their families cannot choose where to seek critical care when ill or which service provider they would prefer; most of the current quality measures used in ICUs are not applicable to adolescents.

However, the themes we have identified have significant overlap with previous research on experience of care indicators for adolescents who are not critically ill [2, 16]. Our participants highlighted the age-appropriateness of the environment, respectfulness and friendliness of staff, communication and inclusion in healthcare decisions as key determinants of perceived quality of healthcare in the ICU.

Unexpectedly, medical competency and health outcomes did not feature prominently in our study. It seems unlikely that a good medical outcome is not important to adolescents and families, but there are other possible explanations for this omission. Adolescents may define characteristics of quality healthcare according to whether the standards they encounter match their expectations [9]—if the standards of care largely met their expectations, they may not have been mentioned in the interviews. Additionally, adolescents may have been unconscious for the more intensively ‘medical’ parts of their admission. Accessibility was not mentioned at all. This is surprising; although in the UK cost is not a barrier to hospital treatment, paediatric intensive care is centralised and travel for patients and families can therefore be significant.

Half of admissions to PICU are for infants under 1 and the majority of the remaining patients are 5 years old or less [10]. In AICU, the majority of patients are 60 years old or older [7]. Adolescents are a minority in each setting. Despite sampling from four intensive care units (two general, two paediatric) over 12 months, only a small number of 14–17-year-old patients met inclusion criteria (emergency admission longer than 24 h) and even fewer were awake for part of their ICU stay. Furthermore, parents of adolescents are more likely than either the elderly or the parents of infants to be in paid work and adolescents often have time taken up by school examinations, thus making it practically difficult to participate in research of this nature. Although this was not explicitly stated by any family declining involvement, an emergency critical care admission is commonly a traumatic experience [14] and families may be reluctant to revisit events as part of a research study. Finally, as critical care services, particularly PICUs, are centralised, families come from a wide geographical area, with a resulting impact on travel time and logistical arrangements for researchers and families.

Despite these difficulties, we were able to begin to gain an understanding of factors of importance to critically ill adolescents and their families. However, in addition to a small sample size, our methodology and patient group introduced a number of limitations. Firstly, families approached to take part were those known to specialist nurses who assisted with recruitment. It is possible that families opting into the study were those who had a better relationship with the nurses at their hospital. Secondly, interviews were conducted with both the adolescent and their parent present; while this served to elicit a more thorough account than if the adolescent had been interviewed alone, it is also possible that participants censored their responses. Thirdly, patients often found it difficult to remember the details of their admission to critical care; in some cases it was evident that their perceptions and accounts had been influenced by their parents. Previously, adolescents and their parents have been shown to have broadly similar but not identical responses to questions on quality of healthcare [4, 8].

Intuitively, it would be expected that the best way to find out how to optimise care for critically ill adolescents and their families is to ask them directly about their experiences. Although this approach has given us some insight into what may be important to them, we have demonstrated that this methodology would not be feasible to scale up to gain the definitive information needed to guide practice change. To optimise care for this group, two further strands of work are required. Firstly, data are needed on influences on the physical outcomes of adolescents following intensive care. Secondly, our findings can be used to inform the development of a patient-reported experience measure to be administered to larger groups of patients and families, including elective admissions to ICU, to gauge the relative importance of the themes identified, and the extent to which they are delivered in different settings. We have reported our methodology previously for developing a patient-reported experience measure for paediatric inpatients and outpatients in a specialist children’s hospital, and a similar mixed-methods, staged approach could be adopted with ICU patients [20].

## Conclusions

Despite the success of adolescent wards in other specialities, the smaller numbers of critically ill adolescents mean that most will inevitably continue to be treated in units designed primarily to meet the needs of older adults or younger children. However we can, and should, continue to learn from the experiences of adolescents to ensure their needs are met in any critical care setting.

## Abbreviations

HDU: High dependency unit
ICU: Intensive care unit
UK: United Kingdom

## References

[1] AAP committee on adolescence (2016). Achieving quality health services for adolescents. Pediatrics.

[2] Ambresin AE, Bennett K, Patton GC, Sanci LA, Sawyer SM (2013). Assessment of youth-friendly health care: a systematic review of indicators drawn from young people’s perspectives. J Adolesc Health.

[3] Bensted R, Hargreaves DS, Lombard J, Kilkelly U, Viner RM (2015). Comparison of healthcare priorities in childhood and early/late adolescence: analysis of cross-sectional data from eight countries in the Council of Europe Child-Friendly Healthcare Survey, 2011. Child Care Health Dev.

[4] Byczkowski TL, Kollar LM, Britto MT (2010). Family experiences with outpatient care: do adolescents and parents have the same perceptions?. J Adolesc Health.

[5] Department of Health (2011) You’re welcome—quality criteria for young people friendly health services. Available at: https://www.gov.uk/government/uploads/system/uploads/attachment_data/file/216350/dh_127632.pdf. Accessed December 7, 2014

[6] Hargreaves DS, Sizmur S, Viner RM (2012). Do young and older adults have different health care priorities? Evidence from a national survey of English inpatients. J Adolesc Health.

[7] Intensive Care Audit & Research Network. Key Statistics from the Case Mix Programme Database 2011–2012. Available at: https://www.icnarc.org/documents/Summary%20statistics%20-%202011-12.pdf. Accessed June 30^th^, 2016

[8] Lindeke L, Fulkerson J, Chesney M, Johnson L, Savik K (2009). Children’s perceptions of healthcare survey. Nurs Adm Q.

[9] Moules T (2009). ‘They wouldn’t know how it feels…’: characteristics of quality care from young people’s perspectives: a participatory research project. J Child Health Care.

[10] Paediatric Intensive Care Audit Network (2017) Annual Report. Universities of Leeds and Leicester. Available at: http://www.picanet.org.uk/Audit/Annual-Reporting/Annual-Report-Archive/ Accessed November 26^th^, 2017

[11] Patton GC, Ross DA, Santelli JS, Sawyer SM, Viner RM, Kleinert S (2014). Next steps for adolescent health: a *Lancet* commission. Lancet.

[12] Playfor S, Thomas D, Choonara I (2000). Recollection of children following intensive care. Arch Dis Child.

[13] Rabiee F (2004). Focus group interview and data analysis. Proc Nutr Soc.

[14] Ratzer M, Brink O, Knudsen L, Elklit A (2014). Posttraumatic stress in intensive care unit survivors—a prospective study. Health Psychol Behav Med: an Open Access J.

[15] Ritchie J, Spencer L, Bryman A, Burgess RD (1994). Qualitative data analysis for applied policy research. Analyzing qualitative data.

[16] Sawyer SM, Ambresin AE, Bennett KE, Patton GC (2014). A measurement framework for quality health care for adolescents in hospital. J Adolesc Health.

[17] Tuckwell R, Wood DLB, Mansfield-Sturgess S, Brierley J (2017). A European Society of Paediatric and Neonatal Intensive Care (ESPNIC) survey of European critical care management of young people. Eur J Pediatr.

[18] The United Nations (1990) The United Nations Convention on the Rights of the Child. Available at: https://downloads.unicef.org.uk/wp-content/uploads/2010/05/UNCRC_united_nations_convention_on_the_rights_of_the_child.pdf?_ga=2.139476915.1078307703.1499598179-866423017.1499598179. Accessed January 18^th^, 2018

[19] Wood DLB, Goodwin S, Pappachan JV, Davis PJ, Parslow RC, Harrison DA, Ramnarayan R (2018) Characteristics of adolescents requiring intensive care in the United Kingdom: a retrospective cohort study. Journal of the Intensive Care Society, in press 10.1177/1751143717746047 PMC6110017 30159012

[20] Wray J, Hobden S, Knibbs S, Oldham G (2017) Hearing the voices of children and young people to develop and test a patient-reported experience measure in a specialist paediatric setting. Archives of Disease in Childhood (EPub ahead of print**)** 10.1136/archdischild-2017-313032 28903950

